# Nutritional aspects and quality of life in gastroesophageal cancer patients that underwent colonic interposition

**DOI:** 10.1093/dote/doaf126

**Published:** 2026-02-02

**Authors:** L Blonk, J Straatman, N J Wierdsma, S S Gisbertz, D L van der Peet, G Kazemier, M I van Berge Henegouwen

**Affiliations:** Department of Surgery, Amsterdam UMC location Vrije Universiteit Amsterdam, De Boelelaan 1117, 1081 HV Amsterdam, the Netherlands; Department of Nutrition and Dietetics, Amsterdam UMC location Vrije Universiteit Amsterdam, De Boelelaan 1117, 1081 HV Amsterdam, the Netherlands; Treatment and quality of life, Cancer Center Amsterdam, De Boelelaan 1118, 1081 HZ Amsterdam, The Netherlands; Department of Upper GI surgery, Portsmouth Hospitals University NHS Trust, Southwick Hill Road PO6 3LY Cosham, Portsmouth, UK; Department of Nutrition and Dietetics, Amsterdam UMC location Vrije Universiteit Amsterdam, De Boelelaan 1117, 1081 HV Amsterdam, the Netherlands; Treatment and quality of life, Cancer Center Amsterdam, De Boelelaan 1118, 1081 HZ Amsterdam, The Netherlands; Treatment and quality of life, Cancer Center Amsterdam, De Boelelaan 1118, 1081 HZ Amsterdam, The Netherlands; Department of Surgery, Amsterdam UMC location University of Amsterdam, Meibergdreef 9, 1105 AZ Amsterdam, the Netherlands; Department of Surgery, Amsterdam UMC location Vrije Universiteit Amsterdam, De Boelelaan 1117, 1081 HV Amsterdam, the Netherlands; Treatment and quality of life, Cancer Center Amsterdam, De Boelelaan 1118, 1081 HZ Amsterdam, The Netherlands; Department of Surgery, Amsterdam UMC location Vrije Universiteit Amsterdam, De Boelelaan 1117, 1081 HV Amsterdam, the Netherlands; Treatment and quality of life, Cancer Center Amsterdam, De Boelelaan 1118, 1081 HZ Amsterdam, The Netherlands; Treatment and quality of life, Cancer Center Amsterdam, De Boelelaan 1118, 1081 HZ Amsterdam, The Netherlands; Department of Surgery, Amsterdam UMC location University of Amsterdam, Meibergdreef 9, 1105 AZ Amsterdam, the Netherlands

**Keywords:** colonic interposition, gastroesophageal cancer surgery, nutritional status, quality of life

## Abstract

The use of a colonic interposition after major esophageal surgery leads to substantial anatomical changes, but information regarding the effects of these changes on functional outcomes is limited. Objective of this study was to evaluate the presence of gastrointestinal symptoms, nutritional aspects, intestinal absorption capacity, and health-related quality of life (HR-QoL) in adult patients after colon interposition. This single-center study consisted of three parts. Part 1 involved a retrospective review of anthropometric data, dietary patterns, and gastrointestinal symptoms in all consecutive patients who underwent colonic interposition between 2010 and 2021 and in whom at least 6 months of follow-up were available. Patients from part 1 who were still alive in 2021 were invited for an in-depth evaluation of dietary intake and intestinal absorption capacity. This included measuring daily fecal losses of energy (kcal), fat (g), and protein (g) over a 72-hour period. The coefficients of fat and protein absorption (CFA and CNA) were calculated. Energy balance (kcal/day) was determined by subtracting fecal energy loss (kcal/day) and daily estimated total energy expenditure (eTEE) from the dietary energy intake (kcal/day). Part 3 assessed HR-QoL prospectively using the EORTC QLQ-C30 and OG-25 questionnaires. All consecutive patients presenting to the outpatient clinic between 2014 and 2021 were asked to complete these questionnaires. In part 1 of this study, 30 patients were included. Symptoms of steatorrhea/diarrhea (65%) and dysphagia (42%) were most frequently reported, and 31% could not cease enteral nutrition via jejunostomy or nasal tube due to weight loss or gastrointestinal symptoms. Ten patients were included in part 2 of this study. Intestinal malabsorption of fat and protein (CFA and CNA <85%) was found in 70% of patients, and 60% of patients had a negative energy balance. HR-QoL was measured in 20 patients. Median global QoL score (EORTC QLQ-C30) was 63 (IQR 50-83) and the OG-25 symptom score 19 (IQR 6.9–36). In conclusion a colonic interposition after esophagectomy is accompanied by gastrointestinal symptoms, intestinal malabsorption, and an impaired QoL. Adequate counseling of patients and follow-up with a multidisciplinary approach to treat gastrointestinal symptoms and correct for intestinal malabsorption is recommended.

## INTRODUCTION

In patients with esophageal cancer who receive treatment with curative intent, the esophagus is removed after neoadjuvant chemoradiotherapy. Gastrointestinal continuity is usually restored by creating a gastric tube. In case reconstruction with a gastric conduit is not feasible, the colon can be used as alternative to restore continuity. The gastric tube is the preferred method to reconstruct the gastrointestinal tract after esophageal surgery because it is technically less challenging, requiring only one anastomosis instead of four, resulting in shorter operative times.^1^ However, if the stomach is not available, segments of the colon can be used as alternative. For example, in patients with previous gastric surgery, concomitant gastric cancer, significant gastric involvement, or when the gastric conduit has failed postoperatively due to complications. In our center, the colonic interposition is preferred over jejunal interposition because of local experience and the fact that no vascular anastomosis is needed. It is already known that reconstruction of the esophagus with a gastric conduit has a major impact on the functioning of the gastrointestinal tract, leading to gastrointestinal symptoms, unintended weight loss, and eating difficulties, which negatively affect patients’ nutritional status and health-related quality of life (HR-QoL).^2^^,^^3^ Reconstructing the gastrointestinal tract by using a segment of the colon instead of a gastric conduit leads to more extensive anatomical changes. Consequently, functional outcomes for patients after colonic interposition are expected to be even more impaired. On the other hand, the absence of gastric acid in the colonic interposition may result in fewer complaints of reflux. Given the low incidence of colonic interposition surgeries, functional outcomes have not been studied extensively.^4–7^ Moreover, studies available describe conflicting results, with some studies indicating an acceptable QoL and other studies showing that QoL is clearly reduced in these patients.^4–7^ The primary aim of this study was therefore to conduct an in-depth assessment of functional outcomes following colonic interposition, encompassing presence of gastrointestinal symptoms, nutritional aspects, intestinal absorption capacity, and HR-QoL.

## MATERIALS AND METHODS

### Study design and patient population

This study was performed at Amsterdam UMC, a tertiary referral center that specializes in complex gastroesophageal cancer surgery, including colonic interpositions. This study comprised three parts conducted within the same patient cohort. The number of patients selected for each part varied, due to the availability of data for different study parts. The first part was a retrospective data collection of nutritional aspects and gastrointestinal symptoms during postoperative follow-up. The second part involved a prospective assessment of dietary intake and intestinal absorption capacity, and the third part consisted of prospective HR-QoL measurements during follow-up. This study was approved by the medical ethics committee (WMO 2019.210 and AMCW19_097), and informed consent was obtained from all patients.

### Part 1—Retrospective assessment of nutritional aspects and gastrointestinal symptoms

All consecutive patients who underwent a reconstruction with a colonic interposition after resection of the esophagus for malign and benign diseases between 2010 and 2021, and in whom at least 6 months of postoperative follow-up data were available in their medical files, were selected from a prospectively maintained database. This database included data on patient characteristics, tumor characteristics, and surgical characteristics. The electronic medical files were studied retrospectively to collect data on patients’ anthropometry, type of patients’ diet, and presence of gastrointestinal symptoms. Preoperative body weight (kg) and Body Mass Index (BMI (kg/m^2^)) were compared to data at 12 months postoperatively and were compared to the definitions of malnutrition of the Global Leadership Initiative on Malnutrition (GLIM).^8^ Anthropometry data of patients who developed recurrent disease within 12 months postoperatively was excluded from this analysis.

### Part 2—Prospective assessment of dietary intake and intestinal absorption capacity

In 2021 all patients that were included in part 1, that were still alive, and in a stable postoperative situation (e.g. at least six months after surgery, recovered from any complication, no clinical signs of recurrent disease, and not dependent on enteral nutrition via tube or jejunostomy) were approached to perform a more in-depth assessment of their dietary intake and digestive functioning (intestinal absorption capacity).

Patients were asked to fill in a four-day weighted nutritional diary. The amount of energy and macronutrient (fat, protein, and carbohydrates) content of the nutritional diaries was calculated based on the Dutch Food Composition Database.^9^ To determine whether patients met their nutritional requirements, the resting energy expenditure (REE) was assessed by using the World Health Organization (WHO) equation for patients with a BMI up to 30 kg/m^2^, and the Harris-Benedict formula was used for obese patients.^10^ The estimated total daily energy requirements (eTEE in kcal/d) were determined by adding 30% to the REE.^11^ The energy balance was calculated as follows: dietary energy intake (kcal/day)-fecal energy loss (kcal/day)-eTEE). A negative energy balance was defined as caloric deficit of ≥10% (as compared to eTEE).

Fecal analyses were performed to determine the intestinal absorption capacity and presence of malabsorption. During the last three days of the four-day nutritional diary, patients collected all their feces in three separate containers. Near infrared spectroscopy (NIRS) was used to determine the caloric value of feces (kcal/day), the daily excretion of fecal fat (g/day), the excretion of fecal nitrogen (g/day) as marker for protein, and the percentage of fecal dry matter (%).^12^ The coefficient of fat absorption (CFA) and coefficient of nitrogen absorption (CNA) were calculated by using the following formula: (dietary intake of macronutrients (g/day)-macronutrient excretion in feces (g/day))/dietary intake of macronutrients (g/day)^*^100.^13^ Healthy individuals have an intestinal absorption capacity >85% and therefore in this study an absorption capacity below 85% was defined as malabsorption and below 75% as severe malabsorption.^13^

### Part 3—Prospective assessment of health-related quality of life

All patients who were included in part 1 of this study and disease-free, were approached to fill in QoL questionnaires. Quality of life was measured prospectively with the EORTC-QLQ C30 questionnaire and EORTC-OG25. The EORTC-QLQ C30 is generic quality of life questionnaire including five functional scales, three symptom scales, and a global health aspects score.^14^ Additionally, this questionnaire contains questions about symptoms that are often reported by patients with cancer (including loss of appetite, diarrhea, insomnia, constipation, dyspnea). The raw data scores were calculated, and all scores were linearly transformed so that scores ranged from 0 to 100.^14^ A higher global health aspect score or functional score indicates a better QoL. A higher score on the symptom scale indicated a higher level of symptomatology. The EORTC-OG25 is a supplementary specific for patients with cancer in the esophagus, stomach, or esophagogastric junction. This questionnaire contains six subscales: dysphagia, eating restriction, reflux, odynophagia, pain and discomfort, and anxiety.^11^ The scoring method of this supplementary is conform the scoring method of the EORTC QLQ-C30. Missing data of both questionnaires was imputed in case no more than 50% of the items on a scale was missing, assuming that the missing items were missing at random and that they would have a value equal to average of the other questions within this scale.

### Statistical analysis

This study represents an observational study of a small cohort. Hence, mainly descriptive statistics were used. Dichotomous variables were denoted as frequencies and frequency percentages. Continuous variables, depending on the distribution, are presented as mean (± SD) or median (range or interquartile range (IQR)). Due to limited sample size available, no statistical tests were performed.

### Outcomes measures of interest

The outcome measures of interest were to describe: (i) changes in postoperative body weight and BMI in the first 12 months postoperatively; (ii) patients’ type of diet; (iii) the percentage of patients with functional or gastrointestinal symptoms; (iv) intestinal absorption capacity and presence of malabsorption; and (v) HR-QoL.

### Surgical procedure and standardized postoperative care path

Preoperatively, patients received Picoprep bowel preparation and started a clear liquid diet two days before surgery. In all patients, a midline laparotomy was performed. The right hemicolon was the colon segment of choice in our hospital, as Bauhin’s valve may prevent reflux. Nevertheless, the definitive choice of colon segment relied on intraoperative vascularization, which was assessed using preoperative CT-angiography scans and, since early 2018, also with Indocyanine Green Fluorescence Angiography (ICG-FA) during surgery.^15^ The right hemicolon was flipped and pulled up through the posterior mediastinum in primary surgeries and retrosternally in secondary surgeries. An end-to-end anastomosis was made between the terminal ileum (or, if not possible, between the cecum/ascending colon) and the esophageal stump. A Roux-en-Y reconstruction was then performed, followed by a colo-jejunostomy and an ileo-colostomy to complete the reconstruction. In all patients, a feeding jejunostomy was placed. A nasal colon decompression tube was also placed to decompress the colonic interposition.

Following surgery, patients were monitored for at least 24 hours in a monitored unit. Total parenteral nutrition via a central venous line was initiated immediately after surgery until enteral nutrition reached a sufficient caloric level. Enteral feeding via jejunostomy was commenced after patients had their first bowel movement. The nasal colon tube was left on free drainage and aspirated six times per day. It remained in place until the fifth postoperative day and was removed if output was deemed acceptable and passage of contrast was confirmed on a barium swallow. Once the nasal colon tube was removed, patients were allowed to start ingesting clear fluids. Their intake was then gradually advanced to a regular diet with guidance from a dedicated dietician.

## RESULTS

**Fig. 1:**
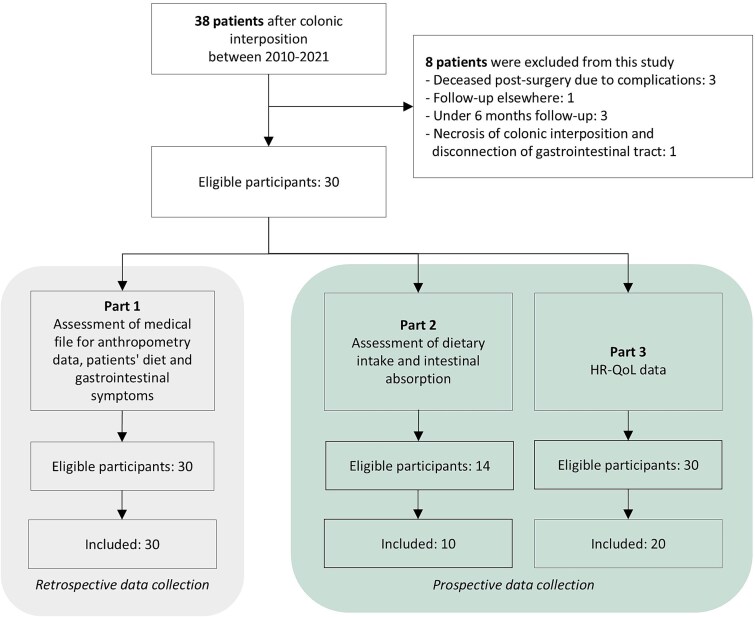
Flow chart of selection of study cohort. (HR-QOL, health-related quality of life.)

Between 2010 and 2021, 38 consecutive patients received a colonic interposition. Figure 1 shows the flowchart of patient selection. The inclusion rate for part 1 of this study was 100%, for part 2 71% and 67% for part 3.

### Patient characteristics

Demographic and clinical data of the three patient groups are summarized in Table 1. The reason for choosing reconstruction with a colonic interposition was failure of the gastric conduit (37%), extent of tumor growth (20%), or previous gastro-esophageal surgery (23%). In most patients, the right hemicolon was used (93%), but in two patients, the transverse colon was utilized due to insufficient vascularization of the right hemicolon after clamping the ileocolic and right colic vessels and arcade along the terminal ileum (as assessed by the macroscopic appearance of the colon and confirmed through perfusion evaluation using ICG). In 7 patients (23%), the postoperative course was uncomplicated, in 15 patients (50%), postoperative complications with a Clavien Dindo score ≥3 occurred, and in 7 patients (23%), a reoperation was needed. Reoperations included correction of diaphragmatic hernia (*N* = 1), anastomotic leakage (*N* = 3), torsion of jejunostomy (*N* = 1), and fascia dehiscence (*N* = 2). Of the 29 patients who were operated for malignant diseases (primary or secondary surgery), 10 patients (33%) developed recurrent disease after a median of 14 months (IQR 9–22) after resection of the tumor.

**Table 1:** Demographic and clinical data of different study cohorts

	Part 1 Retrospective assessment of nutritional aspects and presence of gastrointestinal complaints (complete study cohort)	Part 2 Prospective assessment of dietary intake, intestinal absorption capacity	Part 3 Prospective assessment of HR-QoL
	*N* (%)	*N* (%)	*N* (%)
Participants included in each study part	30	10	20
Female	7 (23%)	3 (30%)	5 (25%)
Age at surgery (years), median (IQR)	64 (59–71)	63 (45–73)	61 (56–69)
Available follow-up (months), median (IQR)	29 (14–53)	48 (19–50)	48 (21–55)
Medical history			
Pulmonary history^*^	6 (20%)	1 (10%)	4 (20%)
Cardiovascular history^**^	12 (40%)	4 (40%)	7 (35%)
Diabetes mellitus (type 1 and 2)	3 (10%)	2 (20%)	3 (15%)
ASA classification ≥3	12 (40%)	5 (50%)	9 (45%)
Reason for reconstruction with colonic interposition			
*Primary colon interposition*			
Extent of tumor growth	6 (20%)	0 (0%)	3 (15%)
Previous gastroesophageal surgery	7 (23%)	2 (20%)	4 (20%)
*Secondary colon interposition*			
Recurrent disease in gastric conduit	1 (3%)	0 (0%)	0 (0%)
Failure of gastric conduit	11 (37%)	5 (50%)	10 (50%)
Fistula after esophagectomy	3 (10%)	2 (20%)	2 (10%)
*Two-stage procedure*			
Incarcerated diaphragmatic hernia	1 (3%)	1 (10%)	1 (5%)
Stomach perforation due to extensive junctional tumor	1 (3%)	0 (%)	0 (0%)
Neoadjuvant therapy^***^			
None	7 (23%)	2 (20%)	5 (25%)
Chemoradiotherapy	19 (63%)	7 (70%)	14 (70%)
Chemotherapy	3 (10%)	1 (10%)	1 (5%)
* Missing*	1 (3%)	0 (0%)	0 (0%)
Type of reconstruction			
Right hemicolon	28 (93%)	10 (100%)	19 (95%)
Transverse hemicolon	2 (7%)	0 (0%)	1 (5%)
Route of reconstruction			
Prevertebral	12 (40%)	2 (20%)	7 (35%)
Retrosternal	16 (53%)	8 (80%)	12 (60%)
Subcutaneously	2 (7%)	0 (0%)	1 (5%)
Clavien Dindo ≥3	15 (50%)	6 (60%)	12(60%)
pT stage			
T0–2	14 (48%)	3 (30%)	9 (45%)
T3–4	15 (52%)	7 (70%)	11 (55%)
pN stage			
N0	22 (76%)	7 (78%)	17 (90%)
N+	7 (24%)	2 (22%)	2 (10%)
Adjuvant therapy^***^	6 (20%)	2 (20%)	1 (5%)

^*^Pulmonary history; including COPD and asthma.

^**^Cardiovascular history; any disease of the cardiovascular system, including hypertension.

^***^Patients in whom the colonic interposition was performed as secondary surgery (i.e. after esophageal resection with gastric tube reconstruction) who received (neo)adjuvant (chemo)radiotherapy before or after their previous esophageal surgery were also included in this group.

Na, data not available.

### Part 1—Retrospective assessment of nutritional aspects and presence of gastrointestinal symptoms

During a median of 29 months (IQR 14–53), patients were followed up at the outpatient clinic. Postoperative anthropometric data at 12 months was available for 18 patients (12 missing), and one patient was excluded from this analysis due to recurrent disease within 12 months after surgery. Median body weight was 70 kg (IQR 58–78) and median BMI was 22 kg/m^2^ (IQR 21–25). At 12 months postoperatively, 9 patients (53%) had lost more than 10% of their preoperative weight and were at risk for malnutrition. Median weight loss in these patients was 11 kg (IQR 9–17). In 1 patient (6%), a BMI <20 kg/m^2^ was identified at 12 months postoperatively. Because 40% of patients had missing anthropometric data at 12 months postoperatively, a sensitivity analysis displaying characteristics for patients with and without anthropometric data available is included in the supplementary material. In patients were anthropometric data was missing 5 patients (42%) had recurrent disease <12 months after surgery.

Data on the type of patients’ diet was available in 29 patients (1 missing). Postoperatively, all patients received total parental nutrition the first days after surgery according to the local postoperative protocol. Among these, 28 patients began enteral nutrition via jejunostomy during their hospital stay, in the patients who discontinued enteral nutrition via jejunostomy, the median duration was 2 months (IQR 1–4). One patient continued total parenteral nutrition due to postoperative complications with the jejunostomy. At the last contact date, 9 patients (31%) were unable to return to their regular diet and remained at least partly long-term dependent on enteral nutrition via jejunostomy due to gastrointestinal symptoms (e.g. dysphagia) or unintended ongoing weight loss. These patients had a median follow-up of 53 months (IQR 30–72). Oral nutritional supplements were prescribed in 14 patients (48%) after enteral nutrition via jejunostomy was stopped. Most of these patients (71%) ceased the usage of oral nutritional supplements after a median of 8 months (IQR 3–16).

In 26 patients (87%), there was documentation available of patients’ visits to the outpatient clinic. In 22 patients (85%), this documentation showed at least one gastrointestinal or functional complaint. Symptoms of steatorrhea/diarrhea (65%, *N* = 17) and dysphagia (42%, *N* = 11) were most often reported. Symptoms of steatorrhea/diarrhea were treated with pancreatic enzymes in 94% (*N* = 16), antidiarrheal medication in 18% (*N* = 3), and antibiotics in 6% (*N* = 1) due to suspected small intestinal bacterial overgrowth (SIBO). Treatments were empirical without prior diagnostic testing for SIBO or exocrine pancreatic insufficiency. Dysphagia was treated with endoscopy with dilatation (64%, *N* = 7), incision therapy (9%, *N* = 1), and Botox therapy (9%, *N* = 1). In 4 patients (36%), no identifiable cause for dysphagia was found with imaging- and endoscopy diagnostics.

### Part 2—Prospective assessment of dietary intake, intestinal absorption capacity

In ten patients, a prospective assessment was done of dietary intake, and intestinal absorption capacity was measured after a median of 18 (IQR 12–24) months postoperatively. Table 2 shows that in 7 patients (70%) there was intestinal malabsorption of both fat and nitrogen (as marker for protein). The median CFA was 79% (IQR 58–84, 95% CI 54–86), and 4 patients (40%) had a severe form of fat malabsorption (CFA < 75%). After excluding one outlier (CFA = 14%), the median CFA increased to 80% (IQR: 65–85%, 95% CI 64–84%). The median CNA was 74% (IQR 60–82, 95% CI 59–82), and in 5 patients (50%), a CNA <75% was identified. Median fecal dry weight was 23% (IQR 20–27).

**Table 2:** Dietary intake and intestinal absorption capacity

Case number	Dietary intake			Fecal analysis					
	Total fat intake (g/day)	Total protein intake (g/day)	Total carbohydrate intake (g/day)	Fat loss (g/day)	CFA (%)	Nitrogen loss (g/day)	CNA^*^ (%)	Fecal weight (g/day)	Dry weight (%)
1	91	66	199	13	86	1.93	82	149	29
2	121	113	292	15	88	2.27	87	243	24
3	115	82	190	20	83	2.60	80	279	23
4	110	64	208	46	58	3.69	64	615	17
5	126	68	263	25	80	3.62	67	340	22
6	166	150	349	38	77	6.07	75	424	26
7	76	62	239	32	57	5.11	48	589	17
8	140	100	348	120	14	9.41	41	1130	21
9	105	69	145	29	72	2.86	74	307	23
10	131	89	708	24	82	1.78	88	198	28

^*^The daily protein intake was converted into nitrogen to calculate the CNA. The following formula was applied: intake of nitrogen (g/day) = intake of protein (g/day)/6.25. CFA, coefficient of fat absorption; CNA, coefficient of nitrogen absorption.

**Fig. 2:**
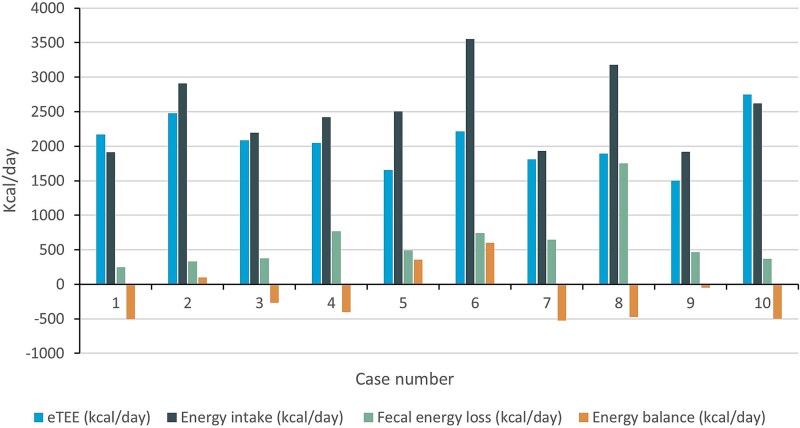
Energy balance. (Kcal; kilocalories; eTEE; estimated total energy expenditure.)

Figure 2 shows the energy balance of these patients. After correcting the dietary energy intake for fecal energy loss, 6 patients (60%) did not achieve their eTEE needs (all in kcal/day). The median energy deficit in these patients was 470 kcal (IQR 269–506).

### Part 3—Prospective assessment of health-related quality of life

**Fig. 3:**
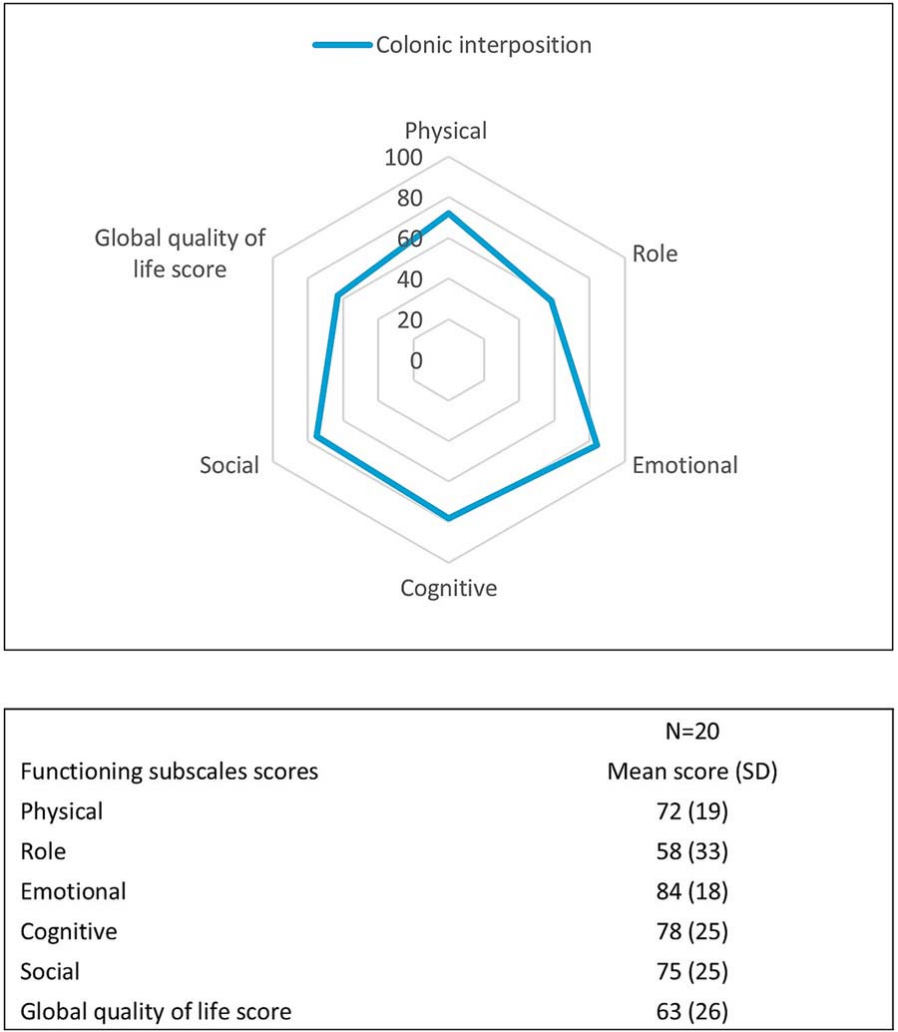
Health-related QoL EORTC QLQ-C30 functional subscales. (SD, standard deviation.)

**Fig. 4:**
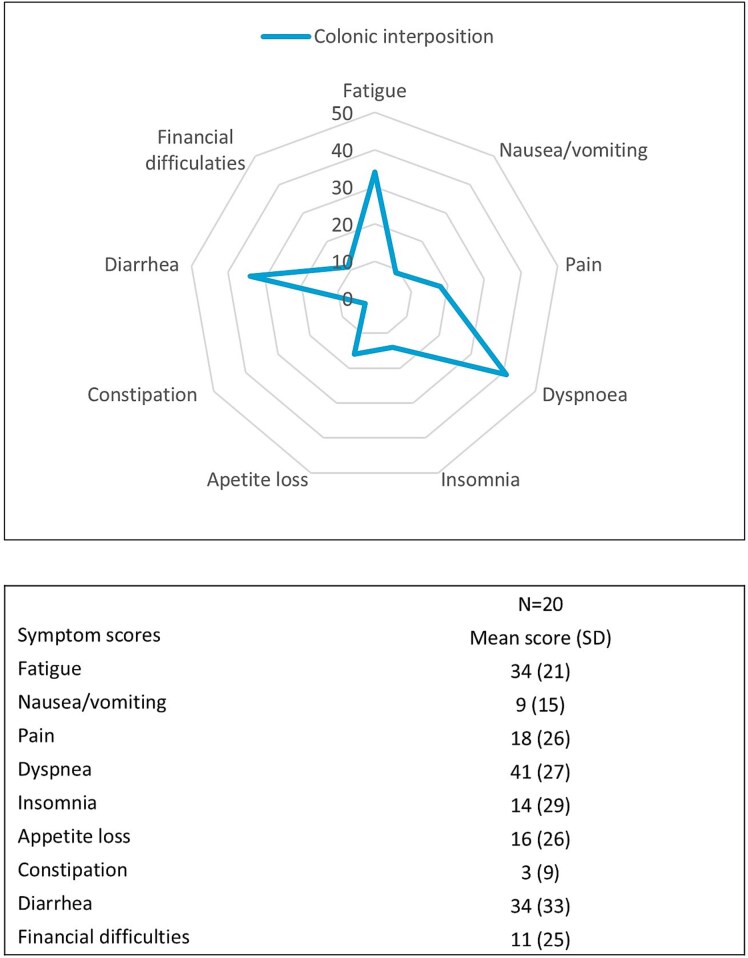
Health-related QoL EORTC QLQ-C30 symptom scores. (SD, standard deviation.)

Health-related quality of life, measured in 20 patients, was determined after a median of 14 months (IQR 8–22) postoperatively. Mean global quality of life score was 63 (SD 26), as presented in Figure 3. Patients after colonic interposition scored highest on the items: fatigue, dyspnea, and diarrhea, indicating more symptomatology (EORTC QLQ-C30, Fig. 4).

**Fig. 5:**
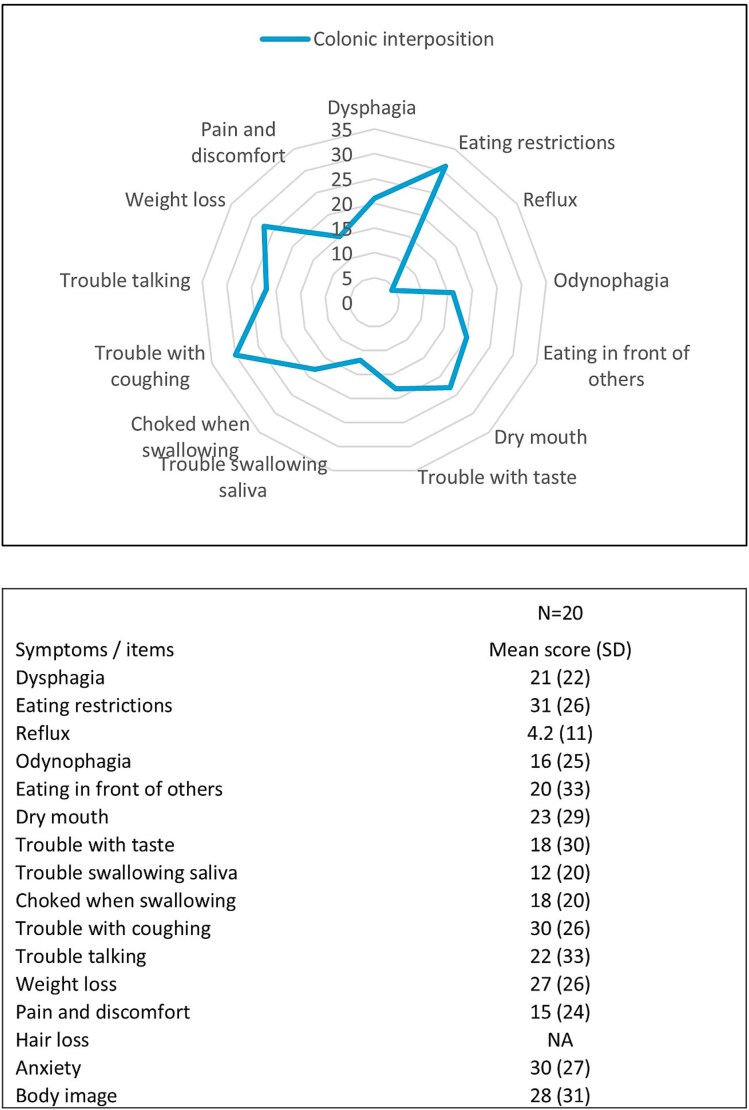
Health-related QoL EORTC OG-25 symptom scores. (NA, not applicable; SD, standard deviation.)

The EORTC OG-25 supplementary shows that patients after colonic interposition scored highest on the following symptoms: eating difficulties, anxiety, trouble with coughing, and weight loss (Fig. 5). The median OG-25 symptom score was 19 (IQR 6.9–36). In one patient within this cohort, a subcutaneously route of reconstruction was performed; this patient had an OG-25 symptoms score of 24, indicating a higher level of symptomatology.

## DISCUSSION

This explanatory study shows that many patients after colonic interposition resection suffer from gastrointestinal symptoms, an impaired intestinal absorption capacity, and reduced HR-QoL. Moreover, a substantial part of patients is long-term dependent on tube feeding or feeding through jejunostomy. These results suggest that the anatomical changes after surgery have great impact on the functioning of the gastrointestinal tract. Postoperative follow-up performed by multidisciplinary teams, including surgeons, dieticians, and gastroenterologists, is recommended to treat symptoms and to prevent deterioration in nutritional status and nutritional deficiencies.

Since the introduction of the gastric conduit, colonic interpositions are only performed by necessity in most centers. Consequently, there is significant heterogeneity between studies and within study populations (e.g. indication for surgery, primary or secondary surgeries, vagal or non-vagal sparing surgeries, route of reconstruction, and duration of follow-up), making it challenging to compare study results.^1^^,^^4^^,^^5^^,^^16^^,^^17^

We found that a significant proportion of patients with a colonic interposition remained long-term dependent on enteral nutrition via jejunostomy, which aligns with some studies but differs from others in terms of the rates of dependence and the ability to tolerate a normal diet.^1^^,^^4^^,^^5^^,^^16^^,^^18^ Although the majority of patients were able to cease enteral nutrition via jejunostomy or tube, 55% of patients in our study cohort reported problems with eating solid foods (EORTC OG-25). A previous study from Coevoet *et al.* had comparable results and found that 30% of patients could not eat a regular diet and were limited to soft and easy-to-chew foods.^16^

Intestinal malabsorption of fat or protein was detected in 90% in this cohort. Most patients already received treatment with pancreatic enzymes or antidiarrheal medication prior to participating in this study, indicating that diarrhea and steatorrhea are known complications. The exact etiology of malabsorption after colonic interposition surgery has not been established yet, but studies performed in patients after esophagectomy (with gastric tube reconstruction) suggest that the following factors may play a role: bile acid malabsorption (BAM), SIBO, a rapid transit time, a shortage of pancreatic enzyme production, and a mismatch between the passage of food and the excretion of digestive fluids contribute to postoperative malabsorption.^19^ As compared to patients after esophagectomy, the occurrence of malabsorption seems higher in patients after colonic interposition.^19^ This study's limited population size prevented determining whether this was caused by the more extensive anatomical changes in colonic interposition surgery compared to a gastric conduit, or that there were confounding factors such as adjuvant therapy, complications from previous surgeries, or comorbidities. Moreover, there was no data in our study on the effect of treatments on these complaints.

Most studies report an acceptable QoL after colonic interposition, and some even suggest superior outcomes compared to patients with gastric tube reconstructions.^1^^,^^5^ Fisher et al. found that the scores of patients with a colonic interposition were not significantly worse compared to the general population after a median of 3 years, and Greene *et al.* found even better scores compared to the general population.^1^^,^^5^ Greene *et al.*, however, only included patients who had a primary colonic interposition and had a median follow-up of 13 years. A follow-up period of 13 years might have allowed patients to adjust to their postoperative changes and challenges. At last, the absence of reflux-related complaints and the lower incidence benign strictures after colonic interposition may lead to a better overall QoL compared to patients after gastric tube reconstruction.^20^ On the other hand Cense *et al.,* reported that patients after colonic interposition scored worse compared to patients after gastric tube reconstruction on the subscales: general health, physical role, vitality, social functioning, and mental health.^4^ Moreover, early satiety, dysphagia, diarrhea, loss of sexual interest, and fatigue were reported most frequently, which is comparable to the results of the here presented study. The cross-sectional design of our study, as well as that of previous QoL studies, limit the ability to assess trends, such as changes in nutritional status or QoL over time, which should be a key focus of future prospective, longitudinal research.

Since colonic interpositions are only performed when necessary and are limited to specific patient populations, it remains uncertain whether these symptoms can be solely attributed to the complex anatomical changes following colonic interposition or if prior treatments received by patients also play a contributing role. Nevertheless, adequate counseling for functional outcomes and QoL of these patients is warranted based on the results of this study. For the past few years, our center has implemented a standardized follow-up schedule, where patients visit the outpatient clinic every six months, alternating between the surgeon and the nurse specialist. A dietician specialized in gastroesophageal cancer surgery is also consulted every 6 months and more frequently if needed. A protocol to uniform treatment of functional complaints has been introduced, and assessment of micronutrients in serum is performed every year.^21^ This protocol includes complaints of dysphagia, reflux, and steatorrhea/diarrhea. Given the high incidence of intestinal malabsorption observed in this study, fecal balance tests should be considered to objectively assess postoperative intestinal absorption capacity. At our center, patients with malabsorption initially receive empirical treatment with pancreatic enzymes. If there is no improvement, other treatments, including antibiotics for SIBO or antidiarrheal medications, are considered. A multidisciplinary team meeting has been established to discuss patients suffering from refractory postoperative functional and gastrointestinal complaints, after failure of treatment suggested in the protocol.

A strength of this study is the thorough assessment of patients’ functional outcomes, including nutritional aspects, intestinal absorption capacity, and HR-QoL. This study, however, has several limitations. Firstly, patients who died postoperatively or had <6 months of follow-up were excluded, potentially introducing survivorship bias and underreporting complications. Secondly, data from part 1 of this study was collected retrospectively, resulting in missing data, particularly for the anthropometric measurements, were 40% of patients lacked 12-month anthropometric data. The possibility that these data are not missing at random could lead to selection bias (sensitivity analysis comparing baseline characteristics is provided in Supplementary table S1). Functional and gastrointestinal symptoms were not documented using standardized forms and relied on patient self-report and physician inquiry, limiting completeness and symptom severity assessment. QoL questionnaires helped address this gap. Thirdly, the small sample sizes in parts 2 (*n* = 10) and 3 (*n* = 20) limit the strength of conclusions and the sample size precluded multivariable adjustment for tumor stage, neoadjuvant treatment, and postoperative complications, any of which might confound the observed associations between colonic interposition and malabsorption or HR-QoL. Therefore, robust conclusions cannot be drawn from these results. Fourthly, the cross-sectional design limits inferences about changes over time in nutritional status or QoL. Finally, empirical treatments for diarrhea and malabsorption were not followed prospectively; therefore, their effectiveness remains unknown. Multi-center, longitudinal studies with detailed peri-intervention monitoring are necessary to address these knowledge gaps. Moreover, all of these single-center findings require validation in multi-institutional cohorts with different surgical volumes and perioperative protocols.

## CONCLUSION

In conclusion, long-term enteral nutrition dependency, intestinal malabsorption, gastrointestinal symptoms, and a reduced HR-QoL were found after gastroesophageal surgery with colonic interposition. These preliminary insights offer perspectives that require further investigation.

## Supplementary Material

Supplementary_table_1_manuscript_coloninterposition_doaf126

## Notes

Financial support: Cancer Center Amsterdam.
