# Supplementary material for: Nutritional aspects and quality of life in gastroesophageal cancer patients that underwent colonic interposition
Source: Dis Esophagus. 2026 Feb 2;39(1):doaf126. doi: 10.1093/dote/doaf126 (PMC12862976; doi:10.1093/dote/doaf126)
Supplement: Supplementary_table_1_manuscript_coloninterposition_doaf126 [file supplementary_table_1_manuscript_coloninterposition_doaf126.docx]

| **Supplementary table 1.** **Demographic and clinical data of patients with and without available anthropometric data** | | | |
| --- | --- | --- | --- |
|  | **Part 1**  **Retrospective assessment of nutritional aspects and presence of gastrointestinal complaints (total group)** | **Patients from Part 1 of this study with available anthropometric data at 12 months** | **Patients from Part 1 of this study with missing anthropometric data at 12 months** |
|  | **N (%)** | **N (%)** | **N (%)** |
| **Participants included in each study part** | 30 | 18 | 12 |
| **Female** | 7 (23%) | 5 (28%) | 2 (17%) |
| **Age at surgery (years), median (IQR)** | 64 (59-71) | 62 (53-72) | 64 (59-69) |
| **Available follow-up (months), median (IQR)** | 29 (14-53) | 49 (22-55) | 14 (8-25) |
| **Medical history** |  |  |  |
| Pulmonary history* | 6 (20%) | 2 (11%) | 4 (33%) |
| Cardiovascular history** | 12 (40%) | 7 (39%) | 5 (42%) |
| Diabetes mellitus (type 1 and 2) | 3 (10%) | 3 (17%) | 0 (0%) |
| **ASA classification ≥3** | 12 (36%) | 9 (50%) | 3 (25%) |
| **Reason for reconstruction with colonic interposition** |  |  |  |
| *Primary colon interposition* |  |  |  |
| Extent of tumor growth | 6 (20%) | 3 | 3 |
| Previous gastroesophageal surgery | 7 (23%) | 4 | 3 |
| *Secondary colon interposition* |  |  |  |
| Recurrent disease in gastric conduit | 1 (3%) | 0 | 1 |
| Failure of gastric conduit | 11 (37%) | 8 | 3 |
| Fistula after esophagectomy | 3 (10%) | 2 | 1 |
| *Two stage procedure* |  |  |  |
| Incarcerated diaphragmatic hernia | 1 (3%) | 1 | 0 |
| Stomach perforation due to extensive   junctional tumor | 1 (3%) | 0 | 1 |
| **Neoadjuvant therapy***** |  |  |  |
| None | 7 (23%) | 3 | 4 |
| Chemoradiotherapy | 19 (63%) | 14 | 5 |
| Chemotherapy | 3 (10%) | 1 | 2 |
| *Missing* | 1 (3%) | 0 | 1 |
| **Type of reconstruction** |  |  |  |
| Right hemicolon | 28 (93%) | 18 | 10 |
| Transverse hemicolon | 2 (7%) | 0 | 2 |
| **Route of reconstruction** |  |  |  |
| Prevertebral | 12 (40%) | 6 | 6 |
| Retrosternal | 16 (53%) | 12 | 4 |
| Subcutaneously | 2 (7%) | 0 | 2 |
| **Clavien dindo ≥ 3** | 15 (50%) | 11 (61%) | 4 (33%) |
| **pT stage** *(1 missing)* |  |  |  |
| T0-2 | 14 (48%) | 9 (50%) | 5 (42%) |
| T3-4 | 15 (52%) | 8 (44%) | 7 (58%) |
| *Missing* | 1 | 1 | 0 |
| **pN stage** *(1 missing)* |  |  |  |
| N0 | 22 (76%) | 15 (83%) | 7 (58%) |
| N+ | 7 (24%) | 2 (11%) | 5 (42%) |
| *Missing* | 1 | 1 | 0 |
| **Adjuvant therapy***** | 6 (20%) | 2 (11%) | 4 (33%) |
| **Recurrent disease <12 months after surgery** | 6 (20%) | 1 (6%) | 5 (42%) |
